# Size variation and geographical distribution of the luminous earthworm *Pontodriluslitoralis* (Grube, 1855) (Clitellata, Megascolecidae) in Southeast Asia and Japan

**DOI:** 10.3897/zookeys.862.35727

**Published:** 2019-07-09

**Authors:** Teerapong Seesamut, Parin Jirapatrasilp, Ratmanee Chanabun, Yuichi Oba, Somsak Panha

**Affiliations:** 1 Biological Sciences Program, Faculty of Science, Chulalongkorn University, Bangkok 10330, Thailand; 2 Animal Systematics Research Unit, Department of Biology, Faculty of Science, Chulalongkorn University, Bangkok 10330, Thailand; 3 Program in Animal Science, Faculty of Agriculture Technology, Sakon Nakhon Rajabhat University, Sakon Nakhon 47000, Thailand; 4 Department of Environmental Biology, Chubu University, Kasugai 487-8501, Japan

**Keywords:** COI, habitat, morphometrics, phylogeny

## Abstract

The luminous earthworm *Pontodriluslitoralis* (Grube, 1855) occurs in a very wide range of subtropical and tropical coastal areas. Morphometrics on size variation (number of segments, body length and diameter) and genetic analysis using the mitochondrial cytochrome c oxidase subunit 1 (COI) gene sequence were conducted on 14 populations of *P.litoralis* from Southeast Asia and Japan. Statistical inference on morphometric data revealed significantly different size variations in the body length and diameter among these 14 populations of *P.litoralis*. However, discordance between the morphometric and mitochondrial COI gene-based phylogenetic analyses was evident, where the size variations in *P.litoralis* showed a different pattern from the COI genetic differences. The update on the current distribution of *P.litoralis* is reported and revealed different aspects of the littoral habitat characteristics between Southeast Asia and Japan.

## Introduction

Earthworms are considered as both ecosystem engineers (Jones et al. 1994) and keystone species (Blondel and Aronson 1995), and they function as decomposers, consumers, and food resources for animals (Lavelle et al. 1992). Earthworms are terrestrial oligochaetes (Annelida, Clitellata), except for a few semi-aquatic taxa, such as earthworms in the family Almidae and *Eiseniellatetraedra* (Savigny, 1826) in freshwater habitats, and *Pontodriluslitoralis* (Grube, 1855), *P.primoris* Blakemore, 2000, and *P.longissimus* Seesamut and Panha, 2018 in marine littoral habitats (Blakemore 2007; Seesamut et al. 2018).

*Pontodriluslitoralis* has a wide distribution in the tropical and subtropical coastal habitats of the Atlantic, Indian, and Pacific oceans. In Thailand, the first record of the littoral earthworm *P.litoralis* was from Khanom District, Nakhon Si Thammarat Province (Panha et al. 2007). Recently, Seesamut et al. (2018) re-examined the littoral earthworms in Thailand and described a new species, *P.longissimus*, based on distinct morphological characteristics and molecular genetic distances from *P.litoralis*. In Japan, the littoral earthworm was first discovered in Matsushima Bay, Miyagi Prefecture and described as *P.matsushimensis* by Iizuka (1898), but later this species was synonymized with the cosmopolitan *P.litoralis* (Easton 1984). Yamaguchi (1953) reported *P.matsushimensis* from Miyakojima in the Miyagi Prefecture, Misaki and Akashi in the Hyogo Prefecture, Ranshima (Hokkaido) and Fukuoka (Kyushu island). Subsequently, the distribution of this species in Japan was been further studied (Ohno 2003), with *P.litoralis* being recorded in more than 20 localities in Honshu, Shikoku, Kyushu, and Ryukyu (Oba et al. 2011, 2015). In addition, the occurrence of *P.litoralis* was also recorded on the beaches of Awaji Island, located between Honshu and Shikoku (Hara et al. 2016). Together, these reports indicate that *P.litoralis* is a cosmopolitan species and occurs in a very wide range of subtemperate and tropical coastal areas (Gates 1972; Easton 1984; Seesamut et al. 2018).

The study of body size can be helpful in identifying earthworm species, as morphometric characters have been represented as one of the keys for confirming their systematic positions (Chang et al. 2007; James et al. 2010). Morphometric analyses, which use mathematical definitions of size and shape, could be used as an addition to other evolutionary analyses, and the results of which could be interpreted in relation to developmental biology and genetics (Klingenberg 2002). Size variation has been studied in many earthworm species, in order to investigate their morphological variation and apply the results towards the identification of the earthworm species. Oboh et al. (2007) reported that populations of the terrestrial earthworm *Eudriluseugeniae* from Lagos, Nigeria were separated into three distinct groups based on the statistical analysis of their morphometric parameters in terms of their body weight, length of clitellum, diameter of posterior and anterior ends, total body length, body size diameter, and total number of segments. In addition, the examination of body size and segment number can be used to separate the terrestrial earthworms *Lumbricusterrestris* and *L.herculeus* into two distinct nominal species, which was also supported by DNA sequence analysis (James et al. 2010). The body size and coloration were also used to separate the *Amynthaswulinensis* species complex into three species (*A.lini*, *A.meishanensis*, and *A.wulinensis*) that were otherwise similar in morphological characters, and this was supported by DNA sequence analysis of the mitochondrial cytochrome c oxidase subunit 1 (COI) gene (Chang et al. 2007).

Many distribution records have reported size variation within *P.litoralis* (Gates 1972; Easton 1984; Seesamut et al. 2018). However, none of the studies have yet revealed whether the size variation indicates different species or only morphological variation within the same species. In addition, it is believed that there is only a single cosmopolitan species (*P.litoralis*), which led us to test this hypothesis based on their size variation coupled with a genetic analysis. The objective of this study, therefore, was to investigate the pattern between the size variations (number of segments, body length and diameter) and genetic (mitochondrial COI) variations in the littoral earthworm *P.litoralis* from 14 populations across Southeast Asia (Thailand, Myanmar, Vietnam, Malaysia, and Indonesia) and Japan. Moreover, we report new data on the distribution and habitat types used by this species.

## Materials and methods

### Field collection, preservation, and identification

**Figure 1.**
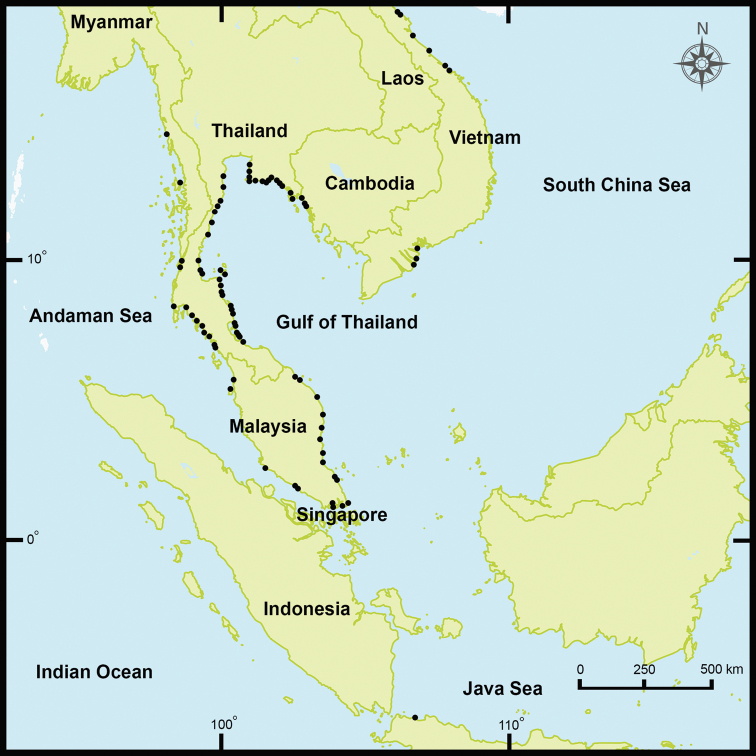
Location and distribution of *P.litoralis* habitats (sampling sites) in Thailand, Malaysia, Myanmar, Singapore, Indonesia, and Vietnam (based on our field collections).

**Figure 2.**
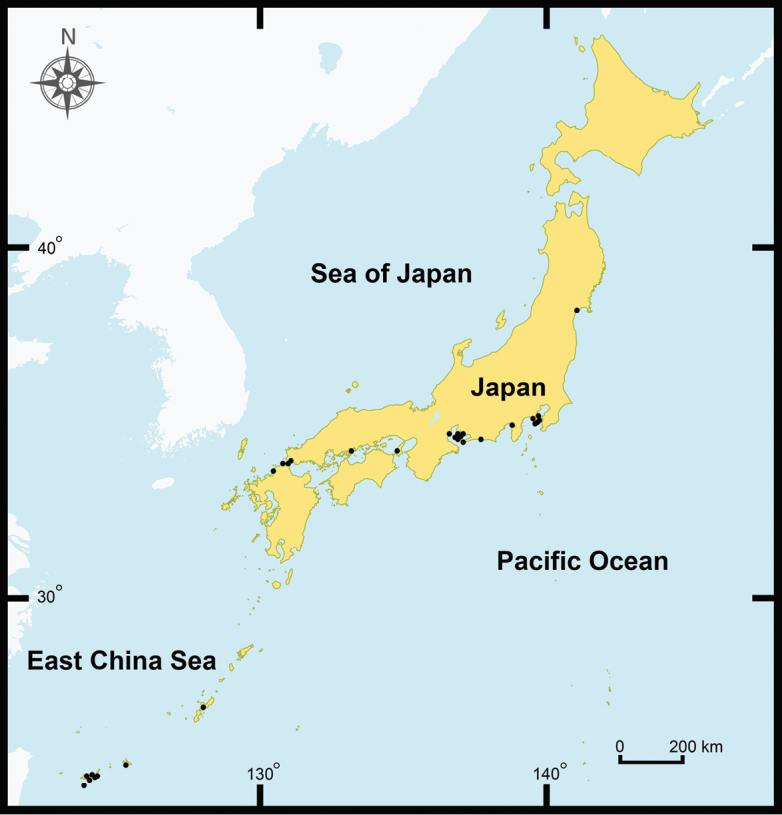
Location and distribution of *P.litoralis* habitats (sampling sites) in Japan (based on our field collections).

From August 2011 to September 2018, samples of *P.litoralis* were collected throughout the coastal areas of both the east and west sides of the Thai-Malay Peninsula (Thailand and Malaysia) and Japan (Honshu, Kyushu, and Ryukyu islands). Moreover, samples from Myanmar, Vietnam, Malaysia, Singapore, and Indonesia were collected (Figs 1, 2). Both adult and juvenile stages of the worms were collected by digging suitable habitats, including sandy beaches at both low- and high-tide levels, estuaries, areas under seaweed debris, damp mud under stones, and areas with wet sand mixed with mud. The living specimens were washed with water, soaked in 30% (v/v) ethanol, photographed, and then killed in 30% (v/v) ethanol. Earthworm specimens were then fixed in 95% (v/v) ethanol for morphological and molecular analyses.

Coordinates of each locality were recorded using a GPS receiver, and salinity values were recorded using an ATAGO refractometer. For species identification, the specimens were carefully identified using the taxonomic literature of Gates (1972), Easton (1984), and Seesamut et al. (2018). Small adults (specimen length <50 mm) and juvenile stages of earthworms were observed under an OLYMPUS SZX16 stereomicroscope. Juveniles were identified by the position of male pores (segment XVIII) showing the inner wall of a longitudinal depression and the internal characters, such as prostate grands on XVIII and absent of nephridia on anterior segments.

### Morphometric analysis

Fourteen populations of *P.litoralis* were selected based on being from different geographic regions (Table 1). At least nine adult worms from each population were then selected and this resulted in a total of 212 specimens used in the morphometric analysis. Only sexually mature earthworms, as determined by the presence of the clitellum, were measured and used to plot the frequency of the length distribution. Total body length, body size diameter, and total number of segments were measured and counted following Ng et al. (2017). Analysis of variance (ANOVA) and principal component analysis (PCA) were performed to assess the significant variation among the three morphometric characters. The mean length and diameter were calculated separately both within each locality and a country scale, and those mean differences were analyzed by one-way ANOVA. The clustering analysis (CA) of the sampling sites was performed to construct a dendrogram depicting the morphological relationship based on the three morphometric measurements, CA were tested based on complete linkage and Euclidean distances. All statistical analyses were performed using the MINITab software v. 18.1 (Minitab, Inc.).

**Table 1.** Sampling localities, GPS coordinates and number of specimens of *P.litoralis* used in the morphometric analysis.

**Locality**		**Latitude, Longitude**	**Number of adult samples**
Thailand (TA)	1. Petchaburi (TA1)	12°49'36.2"N, 99°59'40.3"E	16
	2. Trat (TA2)	12°05'52.4"N, 102°21'27.9"E	20
	3. Chonburi (TA3)	12°50'25.1"N, 100°54'18.3"E	15
	4. Songkhla (TA4)	7°43'30.3"N, 100°22'55.4"E	18
Malaysia (MA)	5. Pulau Pinang (MA1)	5°28'06.7"N, 100°16'41.0"E	16
	6. Pahang (MA2)	3°48'25.0"N, 103°20'29.4"E	18
Myanmar (MY)	7. Dawei (MY1)	14°07'43.5"N, 98°05'50.1"E	10
Indonesia (IN)	8. Banten (IN1)	6°00'51.3"S, 106°40'38.4"E	13
Vietnam (VT)	9. Bến Tre (VT1)	9°48'11.0"N, 106°37'42.2"E	15
	10. Huế (VT2)	16°13'38.9"N, 108°04'58.4"E	16
	11. Nghệ An (VT3)	18°46'06.1"N, 105°45'31.0"E	16
Japan (JP)	12. Aichi (JP1)	34°48'00.2"N, 136°51'30.3"E	18
	13. Hiroshima (JP2)	34°17'45.0"N, 132°19'08.0"E	9
	14. Okinawa (JP3)	26°28'20.0"N, 127°49'54.1"E	12
Total			212

### Molecular analysis

Three specimens were chosen from each of the same 14 populations as in the morphometric analysis resulting in the total of 42 samples used for the molecular analysis (Table 2). The total genomic DNA of each worm was extracted from a posterior body part using a Lysis Buffer for PCR (Takara) DNA extraction kit. The mitochondrial COI gene fragment was amplified using the Tks GflexTM DNA Polymerase (Takara) and the universal primers (Folmer et al. 1994). Each PCR reaction was comprised of 1 μL of Tks Gflex DNA polymerase (1.25 unit/μL), 25 μL of 2x Gflex PCR buffer (Mg^2+^, dNTP plus), 1 μL each of 10 µM LCO1490 (forward) and HCO2198 (reverse) universal primer, 19.5 μL of sterilized distilled water and 2.5 μL of crude lysate (ca 500 ng/μL DNA) with Lysis buffer. Thermal cycling was performed at 94 °C for 2 min, followed by 35 cycles of 94 °C for 1 min, 48 °C for 1 min and 72 °C for 2 min and then a final 72 °C for 5 min. The concentration and quality of the amplicons were determined visually after coresolution through a 1% (w/v) agarose gel against a DNA standard marker in 1x TAE buffer and detected under UV transillumination.

**Table 2.** Details of *P.litoralis* samples using DNA sequencing, and accession numbers of the COI sequences.

**Locality**	**abbreviation**	**GenBank accession number**
1. Petchaburi, Thailand(TA1)	TA1	MK642691
	TA1_A	MK714106
	TA1_B	MK714107
2. Trat, Thailand (TA2)	TA2	MK642690
	TA2_A	MK714108
	TA2_B	MK714109
3. Chonburi, Thailand (TA3)	TA3	MK642689
	TA3_A	MK714110
	TA3_B	MK714111
4. Songkhla, Thailand (TA4)	TA4	MK642688
	TA4_A	MK714112
	TA4_B	MK714113
5. Pulau Pinang, Malaysia (MA1)	MA1	MK642694
	MA1_A	MK714100
	MA1_B	MK714101
6. Pahang, Malaysia (MA2)	MA2	MK642693
	MA2_A	MK714102
	MA2_B	MK714103
7. Dawei, Myanmar (MY1)	MY1	MK642692
	MY1_A	MK714104
	MY1_B	MK714105
8. Banten, Indonesia (IN1)	IN1	MK642698
	IN1_A	MK714092
	IN1_B	MK714093
9. Bến Tre, Vietnam (VT1)	VT1	MK642687
	VT1_A	MK714114
	VT1_B	MK714115
10. Huế, Vietnam (VT2)	VT2	MK642686
	VT2_A	MK714116
	VT2_B	MK714117
11. Nghệ An, Vietnam (VT3)	VT3	MK642685
	VT3_A	MK714118
	VT3_B	MK714119
12. Aichi, Japan (JP1)	JP1	MK642697
	JP1_A	MK714094
	JP1_B	MK714095
13. Hiroshima, Japan (JP2)	JP2	MK642696
	JP2_A	MK714096
	JP2_B	MK714097
14. Okinawa, Japan (JP3)	JP3	MK642695
	JP3_A	MK714098
	JP3_B	MK714099

For sequencing, the PCR products were directly sent to Macrogen Inc. (Japan) without purification. All COI sequences were aligned using the ClustalW algorithm in MEGA7 v. 7.0.18 (Thompson et al. 1994; Kumar et al. 2016) and manually checked by eye. The sequences were aligned, checked and compared with other sequences available in the GenBank databases at the National Center for Biotechnology Information (NCBI), obtained using the BLASTn similarity search tool (http://www.ncbi.nlm.nih.gov). Corrected genetic distances were calculated using the Kimura two-parameter (K2P) model (Kimura 1980) as implemented in MEGA7. Phylogenetic reconstruction was performed using the maximum likelihood (ML) analysis in RAxML v. 8.1.20 (Stamatakis 2014), and 1,000 bootstraps were used to estimate the node reliability as bootstrap support values. Bootstrap values lower than 75% for each node were considered as insignificant (Okanishi et al. 2018). *Pontodriluslongissimus* was used as the outgroup (Accession number MK642683 and MK642684).

## Results

### Size variation of *P.litoralis*

**Figure 3.**
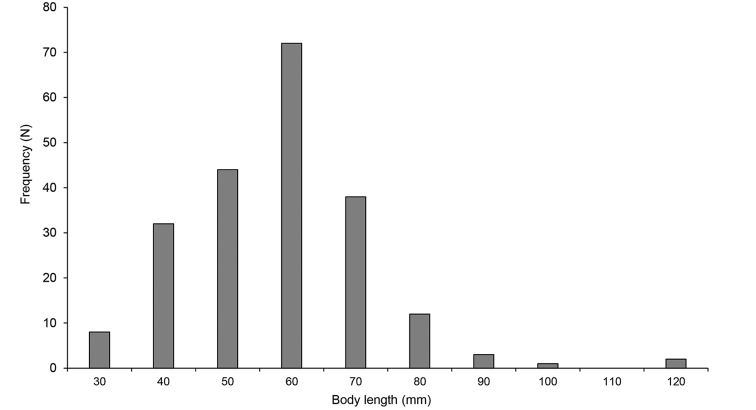
Histogram showing the length frequency distribution of the 212 *P.litoralis* samples from all 14 sampling sites.

**Figure 4.**
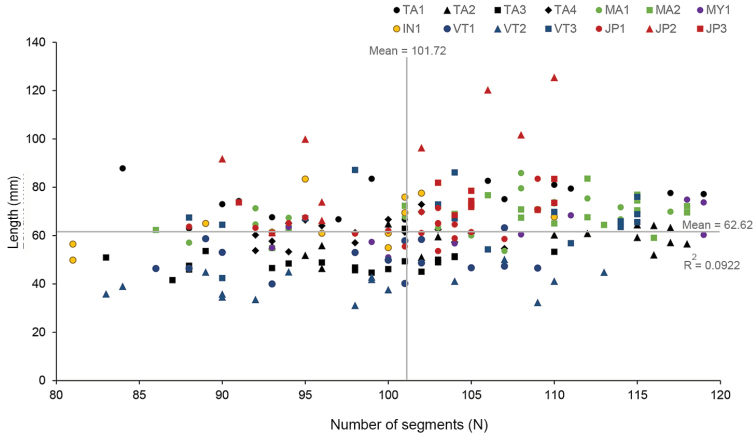
Scatter plot between the length and number of segments of *P.litoralis* (212 samples, 14 locations).

The measurement of 212 individuals of *P.litoralis* earthworms from all 14 sampling sites revealed a length range between 31.1–125.4 mm (Fig. 3). The length between 60.1–69.9 mm occurred at the highest frequency (*n* = 72), followed by that between 50.0–59.6 mm (*n* = 44) and 70.5–79.6 mm (*n* = 38). Two specimens from JP2 were recorded as having a length >120 mm. The relationship between the total number of segments and the body length of *P.litoralis* (Fig. 4), had a low correlation between them (R^2^ = 0.0922). The longest length of *P.litoralis* was 125.4 mm, found in Japan (JP2), while the shortest was 31.1 mm, found in Vietnam (VT2). The mean ± S.D. and median length of *P.litoralis* were 62.6 ± 14.2 mm and 63.1 mm, respectively. The highest number of segments in *P.litoralis* was 119, found in Thailand (TA1) and Myanmar (MY1), while the lowest was 81, found in Indonesia (IN1). The mean ± S.D. and median of the total number of segments of mature *P.litoralis* were 101.7 ± 8.9 and 102, respectively. In addition, the largest body diameter of *P.litoralis* was 4.08 mm, found in Japan (JP2), while the smallest was 1.21 mm, found in Vietnam (VT2). The mean ± S.D. and median *P.litoralis* diameter were 2.12 ± 0.52 mm and 2.02 mm, respectively.

**Figure 5.**
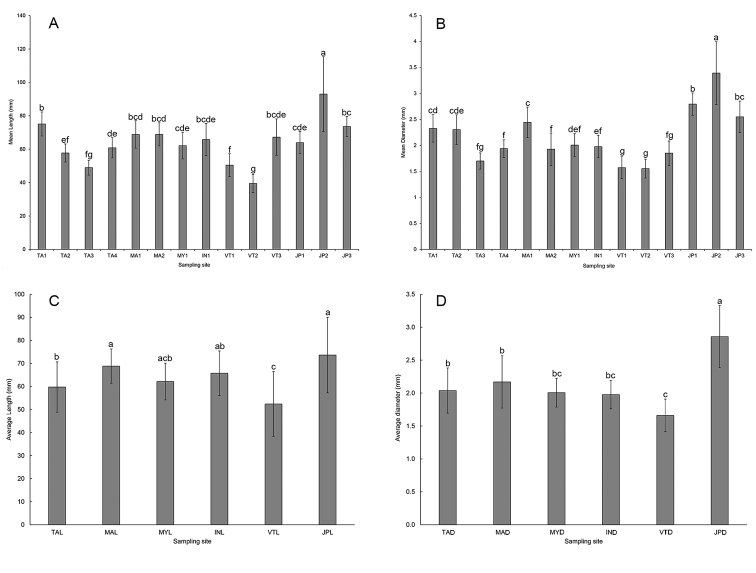
Mean (**A, C**) length and (**B, D**) diameter of *P.litoralis* samples within each (**A, B**) locality and (**C, D**) country sampled in this study. Sampling site codes are given in Table 1. Different letters above the bar indicate a significant difference (*P* < 0.05; one-way ANOVA).

The ANOVA analysis revealed a significant difference (*p* < 0.05) in the mean length and diameter of *P.litoralis* among the 14 locations (Fig. 5). The JP2 population from Japan showed the highest mean body length (93.0 ± 22.4 mm) and diameter (3.39 ± 0.6 mm), while the VT2 population from Vietnam showed the lowest mean body length (39.5 ± 5.4 mm) and diameter (1.55 ± 0.18 mm). Moreover, the highest mean body length (73.6 ± 16.4 mm) and diameter (2.86 ± 0.47 mm) were found in all the Japanese populations (JP1, JP2, and JP3), while the lowest mean body length (52.4 ± 14.2 mm) and diameter (1.66 ± 0.25 mm) were found in all the Vietnamese populations (VT1, VT2, and VT3).

**Figure 6.**
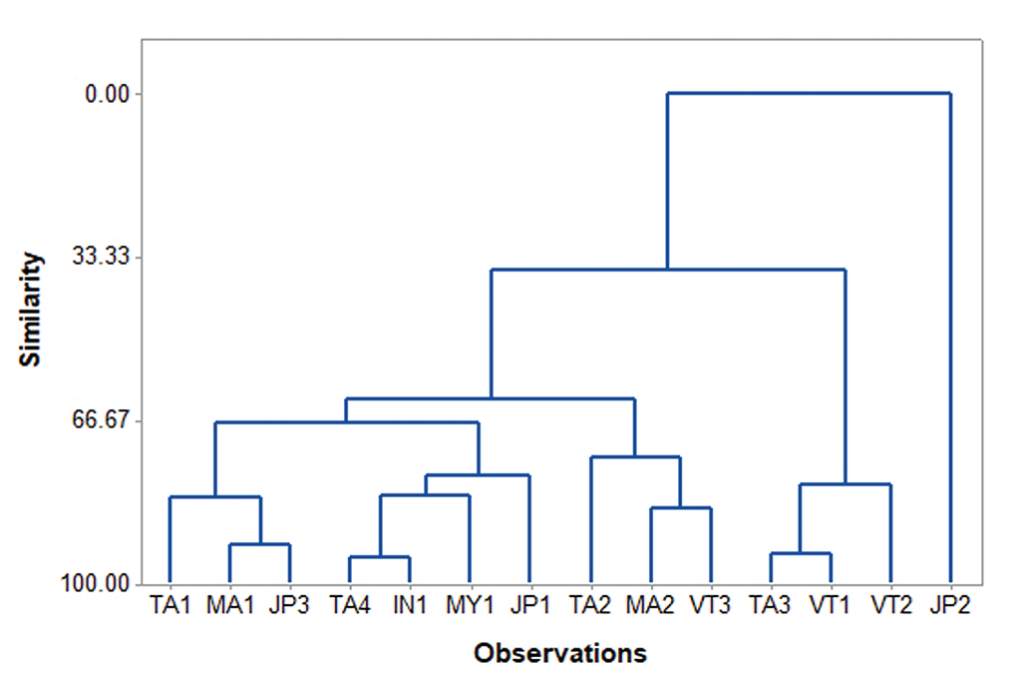
Cluster analysis based on the Euclidean distances among the 14 populations of *P.litoralis*. Sampling sites codes are given in Table 1.

**Figure 7.**
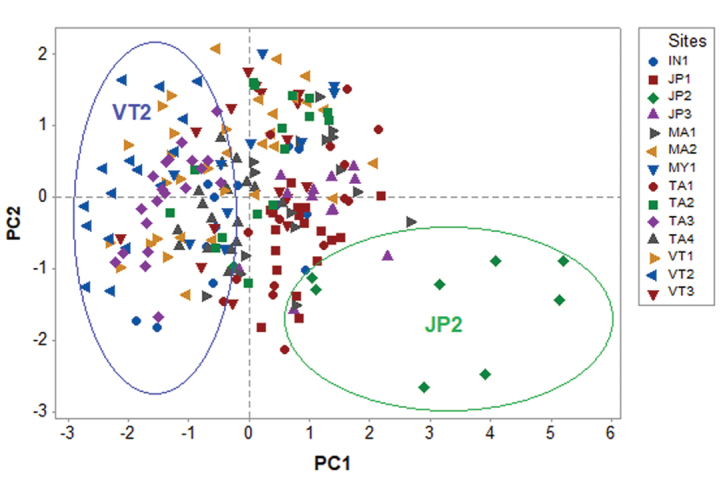
PCA plot between PC1 and PC2 using the three morphometric variables (number of segments, body length, and diameter). Sampling sites codes are given in Table 1.

Cluster analysis of the 14 populations based on the three morphometric data revealed two clusters, one of which contained 13 populations and was further divided into two subclusters, and the other contained only the JP2 population from Japan (Figure 6). The PCA showing the first principal component (PC1) explained more than 60.6% of the variation in the dataset and had a variance (eigenvalue) of 1.8174. The second and third PCs (PC2 and PC3) had a variance (eigenvalue) of 0.8882 and 0.2944, respectively, which accounted for 29.6% and 9.8% of the data variability, respectively. The PC1 revealed that all the loadings were positive, whereas the PC2 showed both positive (number of segment) and negative loadings (body length and diameter). The loadings from the PC2 were less similar among themselves compared to the PC1. The PC1 had a large positive association with the body length and diameter as determined by loadings >0.5, so this PC1 primarily measured the size of the earthworms (Table 3). The scatter diagram of PC1 versus PC2 (Fig. 7) indicated that the size variation within populations of JP2 and VT2 were distinct from other populations.

**Table 3.** PCA percentage of the explained variance and weights of morphometric ratios for the 14 populations of *P.litoralis*.

**Variable**	**PC1**	**PC2**	**PC3**
Length	0.675	−0.143	0.724
Diameter	0.638	−0.380	−0.670
Segment number	0.371	0.914	−0.165
Eigenvalue	1.8174	0.8882	0.2944
% total variance	60.6	29.6	9.8

### Genetic analysis

**Figure 8.**
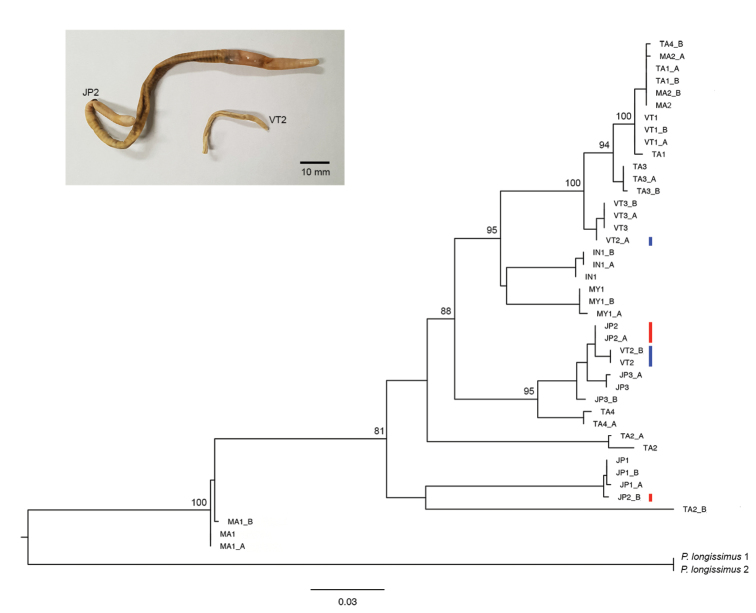
ML phylogenetic tree of *P.litoralis* based on the mitochondrial COI gene (658 bp) with *Pontodriluslongissimus* as the outgroup. Only bootstrap values >70% are indicated at each node. Scale bar represents the number of nucleotide substitutions per site. The sample names correspond to those in Table 5. Photograph on the top left shows comparative size of the shortest and the longest samples in this molecular study. The longest population (JP2) is shown in red and the shortest population (VT2) is shown in blue.

The COI DNA sequences (658 bp) from 42 individuals, three specimens from each of the 14 populations used in the morphometric analysis, were analyzed. The analysis yielded 158 variable (polymorphic) sites and 139 parsimony informative sites. No insertions, deletions, or stop codons were observed in any of the sequences. The K2P genetic distances among the 14 geographical locations within *P.litoralis* ranged from 0.3–12.8 % (Table 4). The highest divergence was estimated between TA1 and JP1; MA2 and JP1 (12.8%), while the lowest was estimated between TA1 and MA2 (0.3%). The genetic distance within group ranged from 0–9%. The highest was estimated in TA2 (9%), whereas the lowest was estimated in VT1 and VT3 (0.0%). The ML tree (Fig. 8) did not show any pattern congruent with the variation in the sizes of *P.litoralis* (Fig. 6). For instance, the analyses on the size variation between the shortest population (VT2) and the longest population (JP2) samples clearly showed a significant difference in their body length (39.5 mm and 93.0 mm for VT2 and JP2, respectively), and body diameter (1.55 mm and 3.39 mm for VT2 and JP2, respectively) (*p* < 0.05), while the cluster analysis confirmed that the two clusters were separated, one contained 13 populations (included VT2) and the other contained only JP2. However, the genetic distance analysis showed a low genetic distance between VT2 and JP2 population (5.6%; Table 4) and the COIML tree suggested a sister relationship between four samples from VT2 population (VT2, VT2_B) and JP2 population (JP2, JP2_A) (Fig. 8). The nucleotide sequences reported of *P.litoralis* in this study are deposited at GenBank under accession numbers as showing in Table 2.

**Table 4.** Between groups mean genetic distances corrected with the Kimura-2 parameter model among the 14 populations of *P.litoralis*. The bold values represent the genetic distance within group. Sampling site codes are given in Table 1.

	IN1	JP1	JP2	JP3	MA1	MA2	MY1	TA1	TA2	TA3	TA4	VT1	VT2	VT3
IN1	**0.002**													
JP1	0.102	**0.001**												
JP2	0.087	0.073	**0.072**											
JP3	0.079	0.108	0.043	**0.011**										
MA1	0.089	0.101	0.103	0.102	**0.002**									
MA2	0.064	0.128	0.100	0.083	0.112	**0.001**								
MY1	0.048	0.109	0.088	0.076	0.109	0.073	**0.002**							
TA1	0.067	0.128	0.099	0.082	0.111	0.003	0.074	**0.005**						
TA2	0.100	0.111	0.112	0.110	0.108	0.119	0.101	0.118	**0.090**					
TA3	0.064	0.125	0.093	0.075	0.106	0.018	0.068	0.017	0.113	**0.001**				
TA4	0.071	0.109	0.074	0.055	0.107	0.065	0.078	0.067	0.114	0.067	**0.067**			
VT1	0.067	0.127	0.098	0.081	0.109	0.005	0.074	0.004	0.116	0.013	0.066	**0.000**		
VT2	0.073	0.113	0.056	0.035	0.103	0.067	0.071	0.066	0.109	0.061	0.060	0.063	**0.050**	
VT3	0.067	0.119	0.088	0.072	0.102	0.030	0.060	0.030	0.105	0.024	0.066	0.026	0.052	**0.000**

### Distributions and habitats of *P.litoralis*

In Southeast Asia, *P.litoralis* was found scattered over the coastal areas in Thailand, Myanmar, Vietnam, Malaysia, Singapore, and Indonesia (Figures 1 and 2). The northernmost sampling site was at Nghệ An Province, Vietnam (18°45'46.1"N, 105°45'23.54"E), whereas the southernmost site was in Bantan, Indonesia (6°00'51.3"S, 106°40'38.4"E). In this study, we reported the first record of *P.litoralis* in Singapore despite only juveniles being collected from the beach in West Coast Park (1°17'45.0"N, 103°45'43.1"E). Among the localities in the sub-tropical areas, *P.litoralis* specimens were collected from various beaches in Japan, and the northernmost site was Matsushima Kaihin Koen in the Miyagi prefecture, where the synonym of *P.litoralis* (*P.matsushimensis*) was originally described from. In total, 29 localities were recorded in the distribution range of *P.litoralis* within Japan, including in Honshu, Kyushu, and the Ryukyu islands.

**Figure 9.**
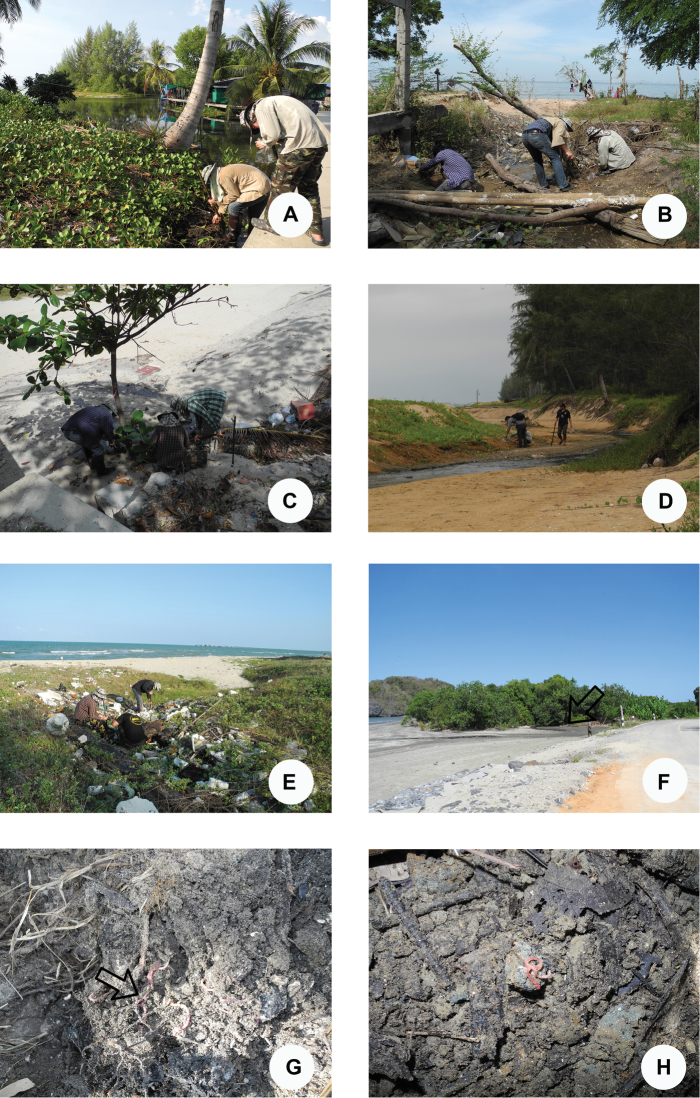
Photographs showing the habitats of *P.litoralis* in Thailand **A** Trat Province **B** Chonburi Province **C** Petchaburi Province **D** Chumphon Province **E** Songkhla Province **F** Satun Province **G** Petchaburi Province **H** Satun Province

**Figure 10.**
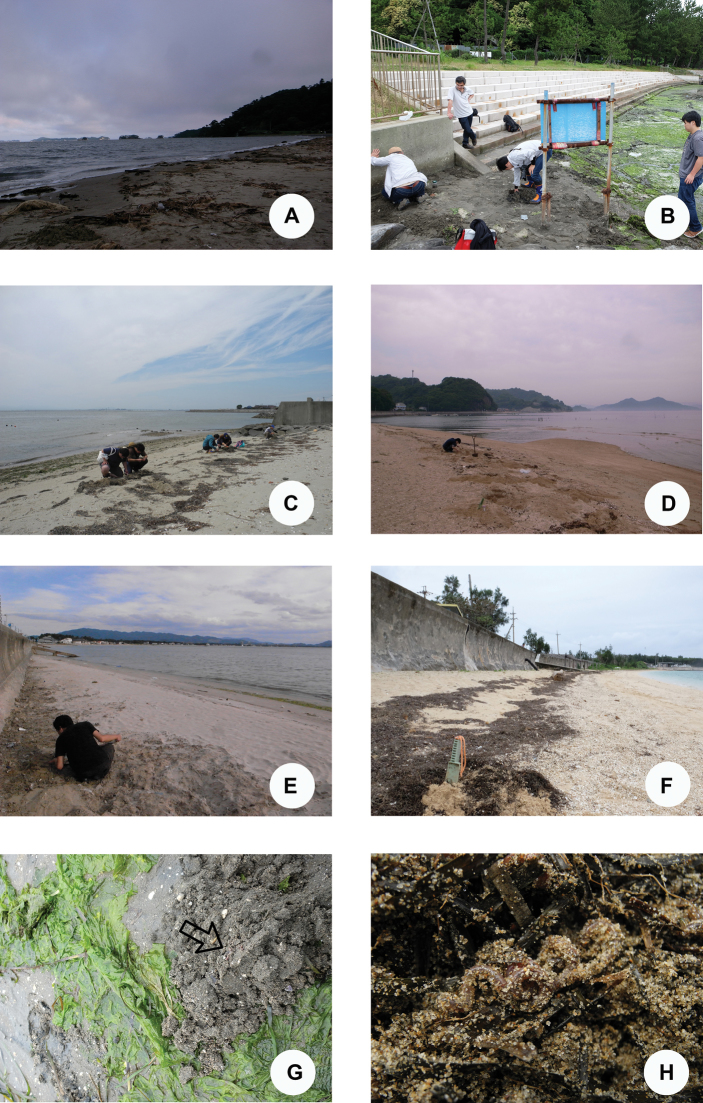
Photographs showing the habitats of *P.litoralis* in Japan **A** Miyagi Prefecture **B** Kanagawa Prefecture **C** Aichi Prefecture **D** Hiroshima Prefecture **E** Fukuoka Prefecture **F** Okinawa Prefecture **G** Kanagawa Prefecture **H** Aichi Prefecture

Based on field collections within Thailand and some parts of Southeast Asia, *P.litoralis* was found to occupy several types of habitats (Table 5; Fig. 9), such as estuaries, brackish habitats, damp mud under stones, under the trash or leaf litter on sandy beaches, mangrove swamps of the intertidal zone, sanitary sewer links, and freshwater channels between the mainland and the sea. However, collections of *P.litoralis* in the Japanese coastal areas showed that *P.litoralis* was abundant and mostly found in sandy beaches facing the ocean and lives in the sand mixed with seaweed debris (Fig. 10). Records of the salinity values during the field collections showed an average salinity between 12–22 ‰ (Table 5).

**Table 5.** Salinity records (mean ‰ ± SD) and habitat characteristics of the sampling sites of *P.litoralis* in this study.

**Locality**	**Collection time**	**Salinity (‰)**	**Habitat**
Thailand	January 2015 – March 2018	19.29 ± 12.14	Salty mud margins of estuaries, brackish lakes, damp mud under stones, mangrove swamps, under the root of the tree near the shore, under the trash or leaf litter on the sandy beach, sanitary sewer emptying to the sandy beach
Myanmar	April 2016	18 ± 12.82	Estuaries, under the trash on the sandy beach
Malaysia	January 2016	15.94 ± 9.85	Estuaries, damp mud under stones and the beach, under the trash or leaf litter on the sandy beach
Vietnam	May 2018 – July 2018	19.38 ± 10.57	Estuaries, under the trash or leaf litter on the sandy beach
Indonesia	August 2017	12	Sanitary sewer emptying to the sandy beach
Singapore	December 2017	22	Under the root of the tree near the shore
Japan	August 2011 – September 2018	17.5 ± 9.85	Sand beach facing to the ocean (sand mixed with seaweed debris), estuaries

## Discussion

This study is the first attempt to integrate morphometric variations and molecular marker analyses together in the cosmopolitan littoral earthworm *P.litoralis*. The specimens investigated in this study were within the variation range previously reported by Jamieson (2001) (body length 32–120 mm, diameter 2–4 mm, and number of segments 78–120) and Seesamut et al. (2018) (body length 28–136 mm, diameter 1–5 mm, and number of segments 76–128).

According to the results of the one-way ANOVA, there was a significant difference in the body length and diameter among specimens from the different geographical sites. In addition, the PCA results supported that length and diameter had a higher influence than the number of segments in the 14 studied populations of *P.litoralis*. However, the phylogenetic tree did not show any congruent pattern with the size variation of the specimens analyzed in this study. For instance, in both the PCA and cluster analysis the longest (JP2) and the shortest samples (VT2) formed separate groups with statistical differences in their size, whereas a low genetic distance between the two samples from each respective population was detected, revealing that the size variation of *P.litoralis* was independent of the genetic (COI gene) differences.

Differences in the body length, diameter, and number of segments have also reported in other earthworms. The terrestrial earthworm *Metaphirepeguana* (Rosa, 1890) from Penang and neighboring states of Malaysia revealed significant differences in their morphometric variations that were not matched by their genetic difference but rather were affected by the type of habitat (Ng et al. 2017). However, Heethoff et al. (2004) reported a strong correlation between the size of *Octolasiontyrtaeum* (Savigny, 1826) earthworms from Germany and Canada and their mitochondrial cytochrome c oxidase II (COII) sequences, showing that small and large individuals were genetically distinct.

This study is a comprehensive report on the occurrence, distribution and habitat characteristics of the luminous littoral earthworm, *P.litoralis*, in the coastal areas of Thailand, Japan (Honshu, Kyushu, and Ryukyu islands), and some parts of Southeast Asia (Myanmar, Vietnam, Malaysia, Singapore, and Indonesia) based on field collections. This survey supported the assumption that *P.litoralis* is widely distributed in subtropical and tropical coastal ecosystems (Gates 1972; Jamieson and Wampler 1979; Oba et al. 2015; Seesamut et al. 2018), and aligns with the worldwide distribution records (Easton 1984; Blakemore 2002).

In general, the distribution of earthworms is mostly affected by environmental factors, such as the temperature, organic matter content, and soil moisture (Johnston et al. 2014). This survey of *P.litoralis* habitats in Thailand and Southeast Asia revealed that the earthworms live in various habitat types with a relatively wide range of salinity and diverse sources of water. The earthworms were mostly found in the ecotone between the terrestrial and marine habitats, such as the mangrove swamps of the intertidal zone, sanitary sewer emptying to sandy beaches, estuaries, salty mud under stones near the shore, and under the trash or leaf litter on sand beaches. This indicated that *P.litoralis* mostly prefers to inhabit the ecotone between terrestrial and marine habitats. The earthworms were found to occupy the soil column that ranged from the top soil down to 30 cm deep, and on humid substrates in contact with tidal seawater, the level of which is an important factor governing the distribution of intertidal species (Penas and Gonzfilez 1983). In this survey, the habitats of *P.litoralis* in Japan, where the worms were collected, were mostly in sand mixed with seaweed debris on the sandy beaches facing the ocean, whereas we did not collect any littoral earthworms from this type of microhabitat in Southeast Asian shores.

In Japan, beach-cast seaweeds have been reported as important habitats and food for a diverse community of marine and terrestrial organisms, such as amphipods, isopods, and copepods (Okuda 2008). The habitats of *P.litoralis* in Japan are similar to those reported in Western Australia coastal areas, where the earthworms were recorded in high density within the wrack material, seaweed, and debris deposited on arid beaches, which provided a rich food resource and resulted in a high abundance of earthworms (Blakemore 2007; Coupland and McDonald 2008). Carlo et al. (2012) reported the preference of *P.litoralis* to inhabit sites with an accumulation of macrodetritic matter that provided abundant organic matter contents and shade that helped to keep the soil surface cool during daytime. Moreover, the salinity of the *P.litoralis* habitats recorded in this study indicated that *P.litoralis* can survive a wide range of salinity between 1–33‰ (Seesamut et al. 2018), the upper bound of which is near the salinity of seawater in general (35‰; Schmidt et al. 2018). Taken together, we suggest that the habitat preference of *P.litoralis* is primarily determined by the abundance of organic matter contents but not the salinity.

In conclusion, although morphometric examinations of size variation could make reliable distinctions among different populations of *P.litoralis*, this distinction was not congruent with the phylogenetic relationship based on COI gene sequence analysis, reflecting that the size variation of *P.litoralis* did not correlate with their genetic (COI) differences. Thus, we propose that the food resource is the key factor underlying size variation in *P.litoralis*. Future analyses on the type of habitats, sand texture, and components of the food resources are necessary. Moreover, studies on salinity tolerance are needed to confirm the habitat preference of this littoral earthworm species.
